# Enhancing B-Cell Malignancies—On Repurposing Enhancer Activity towards Cancer

**DOI:** 10.3390/cancers13133270

**Published:** 2021-06-29

**Authors:** Marta Elżbieta Kasprzyk, Weronika Sura, Agnieszka Dzikiewicz-Krawczyk

**Affiliations:** Institute of Human Genetics, Polish Academy of Sciences, 60-479 Poznań, Poland; marta.kasprzyk@igcz.poznan.pl (M.E.K.); weronika.sura@igcz.poznan.pl (W.S.)

**Keywords:** B-cell lymphoma, B-cell leukemia, enhancer, IGH, IGK, IGL, EBV

## Abstract

**Simple Summary:**

B-cell malignancies are a heterogenous group of lymphomas and leukemias and are the 6th most common cancer-related cause of death. Apart from several oncogenes and tumor suppressors involved in their pathogenesis, recently the role of non-coding, regulatory sequences has been implied. Enhancers are DNA elements controlling gene expression to ensure proper cell development and function. However, the activity of enhancers can be redirected, setting cells on the path towards cancer. In this review, we discuss different mechanisms through which enhancers are exploited in malignant B cells. We also highlight the potential of therapeutic targeting of enhancers as a direction for future investigation.

**Abstract:**

B-cell lymphomas and leukemias derive from B cells at various stages of maturation and are the 6th most common cancer-related cause of death. While the role of several oncogenes and tumor suppressors in the pathogenesis of B-cell neoplasms was established, recent research indicated the involvement of non-coding, regulatory sequences. Enhancers are DNA elements controlling gene expression in a cell type- and developmental stage-specific manner. They ensure proper differentiation and maturation of B cells, resulting in production of high affinity antibodies. However, the activity of enhancers can be redirected, setting B cells on the path towards cancer. In this review we discuss different mechanisms through which enhancers are exploited in malignant B cells, from the well-studied translocations juxtaposing oncogenes to immunoglobulin loci, through enhancer dysregulation by sequence variants and mutations, to enhancer hijacking by viruses. We also highlight the potential of therapeutic targeting of enhancers as a direction for future investigation.

## 1. Introduction

B-cell malignancies are a diverse group of blood cancers which include several types of leukemias and lymphomas: Hodgkin’s lymphoma and non-Hodgkin lymphomas [1,2,3]. They originate from B cells at different developmental stages [4]. Among all cancers, lymphoid malignancies are reported to be the 6th cause of death in the United States [5]. Several factors have been implicated in the pathogenesis of B-cell neoplasms, from genetic mutations, altered miRNA and lncRNA expression to epigenetic changes [4,6,7,8,9,10].

Enhancers are regulatory DNA elements with a pivotal role in shaping cell type-specific transcriptional programs in response to intra- and extracellular signals [11]. They contain sequences recognized by transcription factors and serve as platforms for assembly of an enhanceosome [12]—a multi-protein complex, able to recruit chromatin remodelers and RNA polymerase at the promoter region of target gene, and eventually lead to its expression. Characteristic features of active enhancers include DNase I hypersensitivity indicating open chromatin, presence of binding sites for multiple transcription factors, binding of transcription co-activators and presence of specific chromatin signature marks, such as high histone H3 lysine 4 monomethylation (H3K4me1) but low trimethylation (H3K4me3), and high histone H3 lysine 27 acetylation (H3K27ac) [13]. Enhancers are essential for proper development and functioning of organisms, while their dysregulation might lead to disease, including cancer [14,15].

B-cell neoplasms are a classical example of the enhancer involvement in malignant transformation. The first described eukaryotic enhancer was an intronic Eµ enhancer in the immunoglobulin heavy chain locus [16]. Up to date, several B-cell specific enhancers have been described. Their activity allows for a proper B-lymphocyte differentiation and fulfilling their main function: secretion of high-affinity antibodies [17,18,19]. However, the activity of enhancers can be redirected, setting B cells on the path towards cancer. In this review we discuss different mechanisms through which B-cell enhancers are exploited in malignant cells. In Section 2 and Section 3, we describe the well-studied translocations juxtaposing oncogenes to immunoglobulin heavy or light chain loci. We also discuss mechanisms leading to Ig translocations and the role of Ig enhancers in regulating oncogene expression and malignant development. In Section 4, we present how enhancer dysregulation by germline variants and somatic mutations contributes to development of B-cell neoplasms. Exploitation of enhancers by deregulated transcription factors is described in Section 5. Finally, in Section 6 we focus on enhancer hijacking by certain viruses, showing how B-cell enhancers can be repurposed for viral replication and lymphomagenesis. We also highlight the potential of therapeutic targeting of enhancers as a direction for further investigation.

## 2. Immunoglobulin Heavy Chain Enhancers in B-Cell Malignancies

### 2.1. Structure and Function of IGH Enhancers in Normal B Cells

The *IGH* locus contains several variable (V), diversity (D), joining (J) and constant (C) segments which undergo sequential rearrangements in the course of B-cell maturation to produce the large polypeptide subunit of all classes of immunoglobulins. In the early stage of B-cell development, V(D)J recombination initiated by RAG1 and RAG2 endonucleases brings together one of the different V, D and J gene segments of the *IGH* variable region. Assembly of the recombined VDJ with the Cμ or Cδ constant region results in expression of IgM or IgD molecules, respectively [20]. In mature B cells, antigen-dependent activation triggers somatic hypermutation (SHM) during the germinal center reaction. This leads to further diversification of the variable region of *IGH* and allows selection of B cells with high affinity B-cell receptor. Similarly, upon antigen encounter, class switch recombination (CSR) brings the fused VDJ gene segment in proximity to one of the Cγ, Cε or Cα constant region exons, switching from the expression of IgM/IgD to IgG, IgE or IgA, respectively. SHM and CSR depend on the activation-induced cytidine deaminase (AID) [21,22]. The *IGH* locus contains two enhancers that govern its activity: Eµ and 3′ regulatory region (3′RR).

#### 2.1.1. Intronic Eµ Enhancer

**Figure 1:**
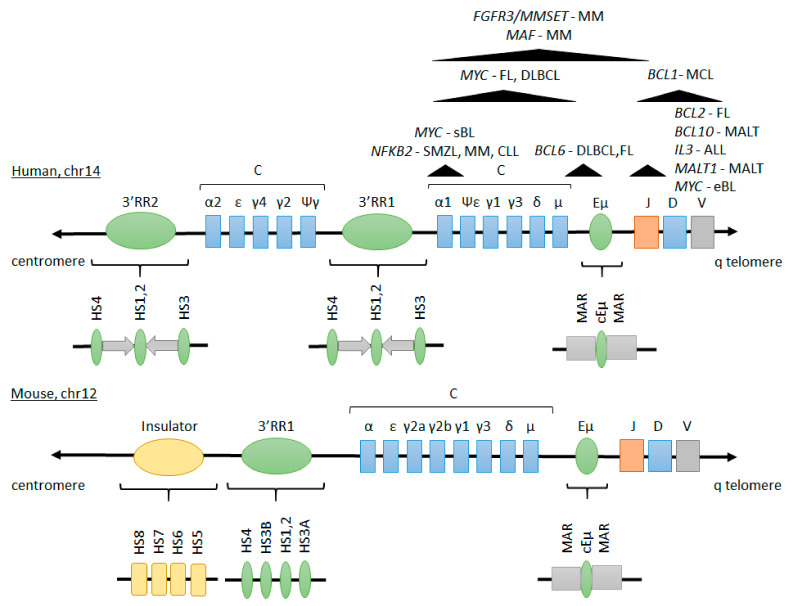
*IGH* locus organization in human and mice. Black triangles mark regions of breakpoints involved in translocations in malignant cells. C—constant region; J—joining; D—diversity; V—variable; HS—DNase hypersensitive site; MAR—matrix attachment region; 3′RR—3′ regulatory region; ALL, acute lymphoblastic leukemia; eBL, endemic Burkitt lymphoma; sBL, sporadic Burkitt lymphoma; CLL, chronic lymphocytic leukemia; DLBCL, diffuse large B-cell lymphoma; FL, follicular lymphoma; MALT, mucosa-associated lymphoid tissue; MCL, mantle cell lymphoma; MM, multiple myeloma; SMZL, splenic marginal zone B-cell lymphoma.

The Eµ enhancer (also known as the intronic enhancer) was the first eukaryotic enhancer described; it was proven to have strong promoter-, distance- and orientation-independent activity in *cis*, specific to B cells [16,23,24,25]. Eµ resides in the intron between the J_H_ region and Cµ exons (upstream to the switch recombination region). It consists of a 220 bp core enhancer element containing sites recognized by multiple transcription factors, flanked by two 310-350 bp matrix attachment regions (MARs) (Figure 1). Control elements within the core enhancer include C/EBP, E1, E5, E2, µA (bound by Ets-1), E3 (bound by TFE3, TFEB, and USF), µB (bound by PU.1), E4, and an octamer sequence (bound by Oct1 and Oct2 together with the specific coactivator OCA-B). Additionally, E2, E4 and E5 are positively regulated by E2A, E2-2, and HEB, in contrast to the negative regulation of E4 and E5 by ZEB (summarized in [26,27]). MARs comprise sites of attachment to the nuclear matrix and contain binding sites for Bright in B cells [28,29], otherwise bound by a negative regulator, Cux/CDP, in non-B cells [30]. Although a limited region containing µA, E3, and µB is sufficient to activate transcription in B cells [31,32], the whole core enhancer element and MARs are postulated to function as locus control region (LCR) [28] and are necessary for efficient transcription of the IGH µ transcript (from V_H_ promoter) [33,34,35].

The intronic enhancer is active throughout B cell development, although especially important in the early stages [27]. It is necessary for the V(D)J recombination—in the absence of its core element, D-J and V-DJ rearrangements are severely impaired [36,37,38,39]. Eµ control of this process is connected to transcription. Prior to D-J recombination, transcription of the Iµ transcript initiates from the Eµ enhancer [40]. At the same time Eµ-dependent D_H_ intergenic antisense transcription starts from the enhancer [41]. The intronic enhancer also promotes histone acetylation in the *IGH* locus before recombination, increasing its accessibility [42]. Moreover, Eμ seems to be responsible for the proper timing of V(D)J recombination, as it initiates the process in pro–B cells but not in pre–B cells [43]. Prior to recombination, the *IGH* locus undergoes radial repositioning and two levels of chromosomal compaction involving formation of multi-looped domains; these processes are also dependent on Eµ [44]. Crucial for the topological alterations are three transcription factors: PAX5, YY1 and CTCF, and the interaction between Eµ and intergenic control region 1 (IGCR1) (reviewed in [45]). Moreover, the Eμ/IGCR1 loop limits RAG1/2 tracking in the first step of V(D)J recombination from the J_H_-related recombination centre (RC) to a domain containing D_H_ and J_H_ gene segments, so the recombination occurs only between D_H_ and J_H_ segments (no V_H_ gene segments) [46]. After V(D)J recombination, Eµ is involved in the checkpoint for allelic exclusion at the pre-B cell to immature B cell transition [47]. The enhancer ensures sufficient Ig µ chain expression required for proper signaling in this process [48].

Studies of the role of Eµ in SHM and CSR initially led to contrary conclusions (reviewed, e.g., in [49]), but it was likely due to the fact that in the absence of Eµ, V_H_ assembly is severely disturbed, which results in the arrest of B cell development. Analysis of mice devoid of Eµ enhancer, but carrying fully assembled V_H_ gene showed that Eµ contributes to both SHM and CSR, yet is not essential for them [50].

#### 2.1.2. The 3′ Regulatory Region

The 3′RR lies downstream of the Cα gene segment and differs between human and mouse [51,52,53,54]. In humans and other Hominoidea (chimpanzee, gorilla, gibbon) 3′RR is duplicated and each region is composed of a 5′→3′ satellite repeat, containing 20 bp tandem repeats, and 3 enhancers: hs3, hs1.2 and hs4. Mouse and rat single 3′RR consist of a 5′→3′ satellite repeat and 4 enhancers: hs3a, hs1.2, hs3b, hs4 as well as 4 insulators: hs5, hs6, hs7, hs8 (Figure 1). In the 3′RR organization, proximal (containing enhancers hs3, hs1.2) and distal (containing hs4) elements are distinguished [53]. Phylogenetic analysis by D’Addabbo et al. showed high sequence similarity of both 3′RRs; 3′RR2 in human being evolutionary older than 3′RR1 [52]. Primate 3′RRs are characterized by the presence of locally repetitive elements with short tandem repeats, similar to switch sequences found in *IGH* locus. On the contrary, in rodents those short tandem repeats are organized in families and are interspaced through the 3′RR palindrome [55]. Hs1.2 is the center of the “quazi-palindrome” flanked by 3 kb inverted sequences, which are conserved in mammals, but not in evolutionarily distant species [52,56,57,58]. The orientation of human hs1.2 enhancers within 3′RR1 and 3′RR2 is also inverted. In mice, hs3a and hs3b enhancers, which are inverted copies of each other, are also part of the palindrome [52]. Preserving the palindromic organization is of key importance for some of 3′RR-controlled functions. Its deconstruction leads to decreased *V_H_* germline transcription, AID recruitment and SHM, while IGH transcription and CSR remain relatively unaffected [52,56,57,58,59]. In humans four allelic variants of hs1.2 have been identified for 3′RR1 and two for 3′RR2 [60,61]. A polymorphism of hs1.2 enhancer is involved in immunological diseases, among others: herpetiform dermatitis, coeliac disease, rheumatic arthritis, diabetes or IgA deficiency [52,62,63,64].

3′RR is often referred to as the master regulator of the *IGH* locus [54,61]. Indeed, it has been implicated in control of majority of recombination events happening at this location [65]. Studies in 3′RR deficient mice revealed that V(D)J is not affected in pre-B cells, supporting the reports that 3′RR activity is obligatory for later developmental stages of B cells [53,65,66]. However, it is speculated that 3′RR might take part in allelic exclusion. 3′RR-mediated inhibition of the *IGH* variable region has been reported, resulting in suppression of V_H_-DJ_H_ recombination. When V(D)J is completed, this effect is abolished [67]. 3′RR is indispensable for SHM and CSR. In B cells from mice lacking 3′RR, heavy chain cannot undergo SHM, while SHM in light chain is not affected [68,69,70]. 3′RR is controlling *IGH* accessibility for AID to enable SHM [58,70]. In order to study 3′RR function in CSR, several knock-out models have been applied [53,70,71,72,73,74]. It has been demonstrated that knocking-out the whole 3′RR significantly impairs CSR, but deletions of single enhancers from 3′RR leave CSR relatively unaffected [58,75]. Combined removal of hs3b and hs4, on the other hand, decreased IGH expression and CSR [71,72,76]. Another study suggested 3′RR involvement in CSR only at its early stages [77]. Interestingly, CSR to IgD was reported as independent of 3′RR regulation [78,79]. Recently, a long non-coding RNA CSR^IgA^ has been identified to interact with hs4 and play an important role in IgA CSR [80].

#### 2.1.3. Interplay between Eµ and 3′RR Enhancers

Although *IGH* enhancers show developmental-dependent manner of activation, they do not act as solitary units. Complex spatial interactions between enhancers themselves, other components of the *IGH* locus and transcription factors were observed [44,54,81,82]. Eµ and 3′RR are separated by ~200 kb and this distance and their spatial relation (3′RR downstream of Eµ) are important for their synergy [83,84]. 3C experiments detected chromatin loop formation between 3′RR and *IGH* variable region [85]. The hs1.2 enhancer emerged as an important player in this interaction. Upon its substitution with Neo^R^, loop formation and IGH transcription were abolished, while Eµ proved dispensable for this interaction. Moreover, hs1.2 quadruplex formation was speculated to regulate transcription factor binding [86]. During CSR, chromatin looping occurs between 3′ RR and Eµ, enabling isotype-specific S-S synapsis formation and possibly reducing the threat of unwanted chromosomal translocations [87,88]. Recent profiling of epigenetic marks and enhancer RNAs (eRNAs) transcription during CSR revealed that in later stages of B cell development, Eµ is actually placed under the 3′RR control. Despite the experimentally confirmed physical association of both enhancers during CSR, Eµ might be dispensable. Its deletion did not affect germline transcription, nor 3′RR epigenetic marks and eRNA expression, while on the other hand deletion of 3′RR reduced transcription rate around Eµ and decreased its H3K9ac [89]. These results further support 3′RR enhancer as the master regulator of the IGH locus.

### 2.2. IGH Translocations in B-Cell Malignancies

The V(D)J recombination and CSR machineries generate several DNA double strand breaks as obligate intermediates, whereas SHM may result in nonmandatory DSBs. These lesions pose a danger of illegitimate recombination outside of the *IGH* locus. The resulting translocations may lead to activation of oncogenes placed under the control of *IGH* enhancers, which is regarded as an early oncogenic hit driving lymphomagenesis. Indeed, several recurrent translocations involving *IGH* have been described in B-cell malignancies. Interestingly, *IGH* translocations occur as well in healthy B-cell populations, which implies that alone they are insufficient to invoke oncogenesis [90,91,92,93]. Likely, genomic instability caused by translocated oncogene deregulation leads to accumulation of other mutations [94]. This is also supported by the in vivo experiments where oncogene overexpression results in malignancy only in a favourable genetic background [95,96].

#### 2.2.1. Mechanisms of *IGH* Translocations

Occurrence of translocations between *IGH* and oncogenes has been mainly attributed to the off-target activity of two key players involved in *IGH* rearrangements: recombination activating gene (RAG) 1 and 2 proteins and activation-induced cytidine deaminase (AID). RAG1 and RAG2 initiate V(D)J recombination in pro-B cells. These lymphocyte-specific endonucleases recognise recombination signal sequences (RSS) of the rearranging segments and cut them exactly between a pair of RSSs and coding sequences. Then, the ends may be additionally modified and finally are ligated by the enzymes of the non-homologous end joining pathway (NHEJ) [97]. However, cryptic RSSs are present throughout the genome and can be processed by RAG [98]. The off-target activity of RAG is determined by various factors, e.g., histone marks, CpG islands or chromatin architecture [46,99,100].

SHM and CSR are completely dependent on AID [101,102] which transforms deoxycytidine into deoxyuridine at the specified sections of Ig loci, inducing error-prone DNA repair. AID displays preference to deaminate cytosine within the WRC motif (where W = A/T, R = A/G), both in vitro [103] and in vivo [104,105], resulting in certain hotspots, influenced additionally by genomic context [106]. Importantly, switch regions contain a double-WRC motif AGCT, in which two adjacent deaminated cytidines lead to double strand breaks in CSR [105,107]. The *IGH* 3′RR enhancer interacting with the Eµ enhancer and appropriate germline transcription promoters of switch regions, brings them together to enable DNA recombination between the S regions [88]. Due to strict regulation, AID activity is mostly restricted to the Ig loci. However, the enzyme also targets a group of actively transcribed genes, including proto-oncogenes like *BCL6*, *MYC*, *CD79A*, *CD79B*, *CD95*, *PIM1*, *MYC*, *RHOH*, *PAX5* [97]. Both hypermutations of those genes [108,109,110,111] as well as their translocations (resulting mostly from erroneous CSR) occur in tumours and in a certain subset of normal B cells [112].

Ongoing transcription seems to be necessary, although not sufficient for the AID targeting and subsequent translocations [113,114]. Two important studies showed that AID especially targets active super-enhancers (SE) and their linked genes [115,116]. In line with this, translocations in lymphomas and leukemias occur either in oncogenes active at some stages of B cell maturation (like *BCL2*, *MYC*) or in genes orchestrating B cell development and activation (e.g., *CD79B*, *PAX5*). Qian et al. also indicated that AID has a preference towards transcriptionally active promoters and enhancers engaged in long-range topological interactions [115], whereas Meng et al. showed that those super-enhancers are characterized by robust convergent sense and antisense transcription [116]. Convergent transcription was shown to increase Pol II stalling, R-loop formation and nascent transcript degradation by exosome [117] which creates single stranded DNA accessible for AID. AID is recruited to the stalled loci via interaction with SPT5 [118]. Accordingly, the breakpoint region in the *IGH/BCL6* translocation is transcribed in both directions: BCL6 from the negative strand and an overlapping lncRNA from the positive strand [119]. Similarly, GRO-seq analysis in ALL patients revealed convergent transcription at the breakpoints [120].

Another mechanism facilitating translocations between oncogenes and the *IGH* locus is their spatial proximity. Early cytogenetic (FISH) studies demonstrated that *IGH*, *IGK*, *IGL* as well as oncogenes loci were preferentially co-localized at certain positions in the nuclei of human B cells [121]. Additionally, smaller distance between the highly translocated sites in comparison to negative controls suggested that their spatial juxtaposition might be a significant factor for the translocation. Together with the advances in cytogenetic and sequencing technologies, more insight into the issue was gained. Hi-C combined with high-throughput genome-wide translocation sequencing in G1-arrested mouse pro-B cells showed that 3D genome organization and spatial proximity significantly influenced genome-wide patterns of chromosomal rearrangements and translocations [122]. The authors proposed a model in which translocation frequency directly depends on the DSB frequency at the two loci and the fraction of cells in a population where they are spatially juxtaposed. The interaction of *IGH* and *c-MYC* loci was studied in more detail. Although Hi-C studies in murine pro-B cells did not reveal specific association of *IGH* and *c-MYC* loci [122,123], such interactions were present in human B lymphoblastoid cells [124,125]. Subsequent studies in mice demonstrated that *IGH* and *c-MYC* are tethered to nucleoli and this increases the frequency of their pairing [126,127]. Spatial conformation of the *IGH* locus in pro-B cells is mediated by CTCF, PAX5 and YY1. In addition, *IGH* and *c-MYC* loci are tethered by CTCF, which may facilitate translocations [128].

#### 2.2.2. Recurrent *IGH* Translocations in B-Cell Lymphoma and Leukemia

**Figure 2:**
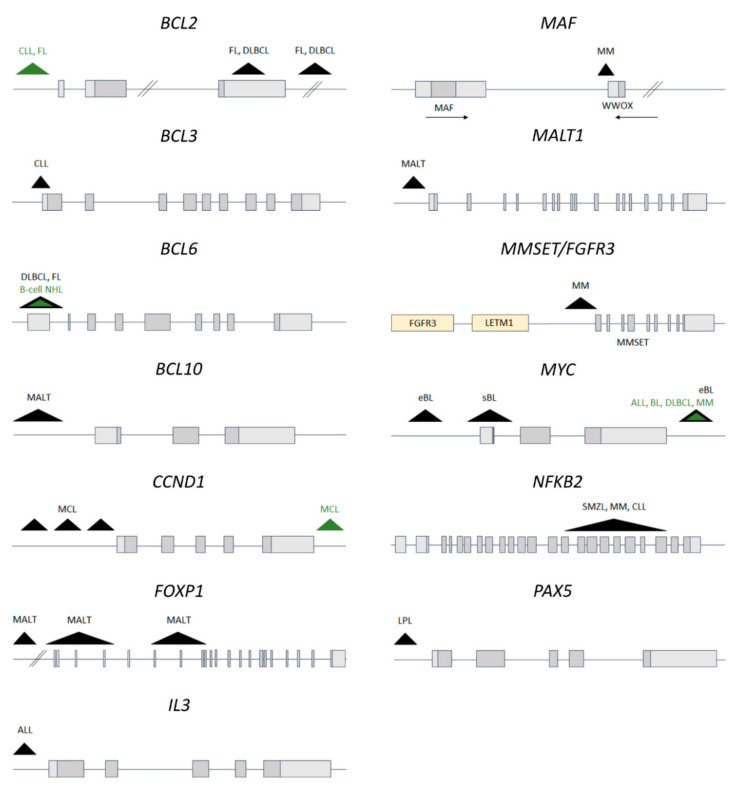
Localization of breakpoints in genes involved in translocations with Ig loci. Black triangles mark regions of breakpoints involved in translocations with *IGH* locus, green triangles—*IGK* and *IGL* loci. Lines depict introns, light grey boxes—noncoding exons, dark grey boxes—coding exons, yellow boxes—whole genes. Genes are oriented 5′→3′ unless indicated otherwise with arrows. ALL, acute lymphoblastic leukemia; eBL, endemic Burkitt lymphoma; sBL, sporadic Burkitt lymphoma; CLL, chronic lymphocytic leukemia; DLBCL, diffuse large B-cell lymphoma; FL, follicular lymphoma; MALT, mucosa-associated lymphoid tissue; MCL, mantle cell lymphoma; MM, multiple myeloma; NHL, non-Hodgkin lymphoma; SMZL, splenic marginal zone B-cell lymphoma.

Translocations between the *IGH* locus and proto-oncogenes are common events in B-cell malignancies. Some translocations are highly prevalent in certain types of neoplasms and are used as diagnostics and prognostic markers. Localization of the breakpoint within *IGH* reflects the developmental stage of a B cell at which the translocation occurred: breakpoints in the variable region happening in pro- or pre-B cells, while breakpoints in the switch region originating from more mature germinal center B cells (Figure 1). On the other hand, sequence motifs at which the translocations happened disclose the enzyme engaged (RAG or AID) [129]. Depending on the translocation partner, different cellular pathways are activated (most often promoting proliferation or inhibiting apoptosis) that ultimately lead to malignant transformation (Figure 2). However, additional genetic hits are necessary for the disease onset. In addition, in a subset of so called double- or triple-hit B-cell lymphomas concurrent translocations involving *MYC* and *BCL2* and/or *BCL6* occur. These tumors are highly aggressive and respond poorly to standard therapies [130]. *IGH* translocations associated with B-cell malignancies have been described comprehensively previously [131], here in Table 1 we indicate the most common ones together with the implications arising from the features of translocated genes.

### 2.3. Role of IGH Enhancers in Regulating Oncogene Expression and Malignant Development

Our knowledge of the precise roles of particular Ig heavy chain enhancers in different steps of B-cell maturation is rather well established. Occurrence of *IGH* translocations in B-cell malignancies prompted studies on the role of *IGH* enhancers in lymphoma. Since Eµ and 3′RR are important regulators of the *IGH* locus activity throughout the B-cell lifetime, the intuitive questions to ask are: if and how can they be implicated in expression of translocated oncogenes? Mouse models of chromosomal translocations, juxtaposing oncogenes with Eµ and/or 3′RR allowed to build our current understanding of their engagement in B-cell malignancies [66,189,190,191]. Three main study approaches can be distinguished: (1) regulation by Eµ; (2) regulation by 3′RR and (3) regulation by both Eµ and 3′RR, the most resembling endogenous conditions. When choosing the mice model, main window of activity of each enhancer should also be kept in mind. Lymphomas developed in mice with an oncogene under regulation by Eµ only represent immature B-cell stage, while stimulation by 3′RR-only results in mature B-cell malignancies [189,192]. Animal models are important not only because they allow to understand the mechanisms driving oncogene expression and malignant transformation, but also provide an in vivo system for testing therapeutic approaches [193]. Therefore, mimicking the translocations is of key importance. It has been observed though, that even if the translocation is present, the development of lymphoma can be variable [193]. This indicates that other factors, besides translocation itself, play a role in lymphomagenesis. Up to date, several mouse models with *IGH* translocations have been established (Table 2).

Eµ-myc mice have been so far the most widely used model [199], reviewed recently in more details by Ferrad et al. [189]. It employs a construct in which Eµ enhancer is placed 5′ to exon 1 of *c-Myc*. Arising lymphomas represent mainly immature B-cell stages. Another knock-in model, iMycEµ, imitates endemic Burkitt lymphoma with *MYC-IGH* translocation t(8;14) in humans/t(12;15) in mice [200,201,206]. Here, *c-Myc* is under the regulation of both Eµ and 3′RR. iMycEµ helped to reveal an aberrant regulatory network involving PI3K, NF-κB and STAT3, important for Myc expression and tumor development, although the involvement of enhancers is not discussed in this work [201].

In contrast to Eµ, 3′RR contains several enhancers. Which of them are of key importance for translocated oncogene expression? Kovalchuk et al. showed that hs3a and hs1,2 enhancers are important drivers of Myc overexpression in mouse plasmacytomas, while hs3b and hs4 are dispensable [204]. Another study indicated that 3′RR is not obligatory for translocated c-Myc expression in pro-B cell lymphomas, but essential in peripheral B-cell lymphomas [205].

Several knock-in models placing *c-Myc* under control of 3′RR enhancers have been developed. Those include: IgH-3′E-myc knock-in mice, iMycCα, iMycCµ and the use of “minimal 3′RR” (also reviewed in [189,190]). The first approach utilizes introduction of murine 3′RR DNase I hypersensitive sites into the endogenous *c-Myc* locus [202]. Even though other *IGH* regulators were not involved, transgene insertion resulted in elevated c-Myc expression and led to Burkitt lymphoma-like malignancy. Although this model clearly demonstrated the ability of 3′RR to deregulate oncogene expression, it does not resemble the native organization of translocation in BL, where exons 2–3 of *c-Myc* are inserted into the *IGH* locus. To further validate the involvement of 3′RR in oncogene deregulation, the minimal 3′ locus control region (LCR) transgene was developed, consisting of *c-Myc* with its P1 and P2 promoters fused with a fragment containing only the core 3′RR sequences: hs3a, hs1.2, hs3b and hs4 [203]. Authors reported increased c-Myc levels and appearance of BL-like cells at 34 weeks of age in animals bearing the transgene.

Recent study by Ghazzaui et al. revealed that 5′ and 3′ *IGH* enhancers cooperate in the induction of B-cell lymphomas [191]. Authors compared three commonly used, previously mentioned, mice models: iEµMyc, iMycCα and iMycCµ. They highlighted the elevated rate of lymphomagenesis and Ki67 index in animals with both Eμ and 3′RR enhancers present and the oncogene placed upstream of Eu (iEµMyc). This model resembles most closely BL cases. iEµMyc mice are characterized by shorter life expectancy and higher c-myc expression levels than other two models. Surprisingly, in iMycCµ, where Eµ is knocked-out, a specific group of B-cell lymphoma cells was reported—a CD19-negative population. The reason of this remains an open question. In iMycCα mice the oncogene is placed among Cα exons, and the Eµ enhancer remains intact [207]. In both iMycCµ and iMycCα mice elevated Myc expression was confirmed and they developed lymphoma, although the onset was delayed compared to the iEµMyc animals.

Apart from Myc, mouse models have been also developed for other oncogenes involved in IGH translocations. In Igh-3′E-bcl2 mice, which aimed to mimic human lymphoma with t(14;18)(q32;q21), 3′RR enhancers were inserted 3′ of *Bcl2* and led to increased mRNA and protein levels [194]. Moreover, *Bcl2* promoter change from P1 to P2 occurred, similarly to native follicular lymphoma cases. Chromosome conformation capture experiments revealed interaction of 3′RR with *Bcl2* locus in Igh-3′E-bcl2 mice, however the exact hs site involved in this interaction was not discussed. Similar interactions were observed in t(14;18) human cell lines. In addition, chromatin immunoprecipitation in human SU-DHL-4 cells revealed OCT-2 and BOB-1 binding to hs1.2 and hs4 enhacers [208]. Interestingly, OCT-1, OCT-2 and BOB-1 were found at promoter 2 of *BCL2*, even though this region does not contain their binding sites.

In another study CCND1-3′RR mice, mimicking human t(11;14)(q13;q32), were created to investigate mantle cell-like and myeloma-like phenotype [196]. Surprisingly, juxtaposition of cyclin D1 with 3′RR was not itself sufficient for malignant transformation. Eµ-cyclin D1 mouse model obtained similar results, but when crossed with Eµ-myc mice, lymphoma occurrence was rapid [197]. This further supports the observation, that other factors besides single translocation are required to drive carcinogenesis.

Eμ c-Maf TG mouse model was developed to study human t(14;16)(q32;q23) found in multiple myeloma [198]. Elevated levels of c-Maf mRNA and protein were confirmed in those transgenic animals, as well as 28% incidence of lymphoma. Transgenic animal models of other chromosomal translocations found in human lymphomas include also: Eµ-BCL10 mice to mimic t(1;14)(p22;q32) [96] or tet-o-BCL6 crossed with Eμ-tTA to study t(3;14)(q27;q32) [195], but those in vivo studies were more focused on investigation of molecular and physiological effects of aberrant oncogene expression than on pinpointing *IGH* enhancers function in malignant transformation.

Despite an important progress in elucidating the involvement of *IGH* enhancers in oncogene expression and lymphomagenesis achieved with the use of transgenic mice, the precise mechanisms still remain to be determined. It should also be kept in mind that besides clear homology between human and murine *IGH* loci, there are a few differences in their organization. Human 3′RR is duplicated, it contains only one hs3 enhancer and lacks hs5-8 insulators. Those differences may limit direct translation of findings from mouse models to humans.

## 3. Immunoglobulin Light Chain Enhancers in B-Cell Malignancies

### 3.1. Structure and Function of lGK and IGL Enhancers in Normal B Cells

**Figure 3:**
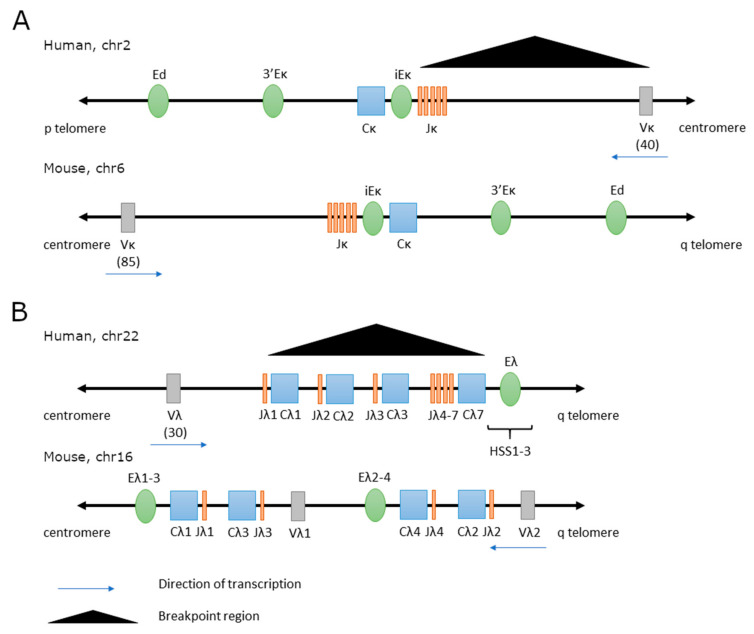
Organization of the human and murine *IGK* (**A**) and *IGL* (**B**) loci. Numbers below Vκ and Vλ indicate the number of variable gene segments. Blue arrows depict the direction of transcription. Black triangles mark regions of breakpoints involved in translocations in malignant cells.

In a subset of B-cell malignancies, the immunoglobulin light chain loci—kappa and lambda—are involved in oncogenic translocations. The *IGK* locus contains three enhancers: the intronic enhancer (iEκ) located between the *IGK* joining and constant genes, and two enhancers localized 3′ of the *IGK* locus, the proximal (3′Eκ) and distal (Ed) enhancer (Figure 3A). Functions of these enhancers have been studied in mouse models, and their genomic organization and sequence of their key elements is strongly conserved across mammals [209]. This suggests that mechanisms of *IGK* gene expression and rearrangements regulation by *IGK* enhancers are similar in human. During B cell development, *IGK* recombination is preceded by profound changes in chromatin structure organization and transcription factor occupancy within the *IGK* enhancers [210,211,212,213]. Moreover, iEκ is critically involved in maintaining the timing of *IGH* and *IGK* rearrangements: V(D)J recombination in *IGH* takes places in pro-B cells and only after it is stopped, recombination in *IGK* can be initiated in pre-B cells [43,214]. All three *IGK* enhancers interact with each other in active *IGK* loci to promote transcription and rearrangements [213,215,216,217], and their activity strongly depends on NF-κB binding to iEκ [215]. In human and mice expression of IGL and IGK is mutually exclusive. Rearrangements are initiated in the kappa locus and in case they are non-productive, the lambda locus is activated. Similar to *IGH* and *IGK* loci, rearrangements and expression of *IGL* genes are also regulated by enhancers [218,219]. There are marked differences between the murine and human *IGL* enhancers. While there are two enhancers in mice: Eλ_2-4_ downstream of Cλ4 and Eλ_3-1_ downstream of Cλ1 [220], human *IGL* locus contains one enhancer downstream of Cλ7 [221] (Figure 3B). Moreover, activity of the human but not mouse *IGL* enhancer strongly depends on NF-κB. At the same time, murine *IGL* enhancers are much weaker than human enhancers and this may be due to a mutated NF-κB binding site whose restoration increases activity of murine enhancers [222].

### 3.2. IGK and IGL Translocations in B-Cell Malignancies

Given the crucial role of *IGK* and *IGL* enhancers in immunoglobulin light chain rearrangements and expression, it is not surprising that translocations juxtaposing light chain enhancers with oncogenes are found in B-cell malignancies, although less frequently than *IGH* translocations. Translocations of *MYC* to *IGL* [t(8;22)(q24.1;q11.2)] and *IGK* [t(2;8)(p11.2;q24.1)] have been described in several types of B-cell malignancies, such as BL, DLBCL, B-ALL and MM [223,224,225,226,227]. Unlike in the case of rearrangements with *IGH*, the breakpoint within *MYC* locus was localized up to 600 kb 3′ of *MYC* (Figure 2). As a result of the translocations, *MYC* was brought in the neighborhood of the *IGK* (up to 50 kb away) and *IGL* enhancers (100–300 kb away). Analysis of the chromatin organization in the BL cell line LY66 bearing the *IGK/MYC* translocation revealed that the physical distance between *MYC* and *IGK* was much shorter than expected for a linear distance [228]. This implies existence of a chromatin architecture allowing spatial interaction between *IGK* enhancers and *MYC*.

A comprehensive study of nearly 800 multiple myeloma patients revealed a wide repertoire of translocations, with 41% involving *IGH*, 10%—*IGL*, and 5%—*IGK*. *MYC* was juxtaposed to *IGH* and *IGL* with the same frequency, and was the most prevalent partner of *IGL* translocations (41%). *IGL* translocations were often accompanied by focal amplifications involving the *IGL* enhancer. Strikingly, patients with *IGL* translocations had worse outcome compared to patients with *IGH* and *IGK* translocations, despite similar levels of MYC expression. The authors propose that this phenomenon might be explained by high levels of IKZF1 bound to *IGL* and thus a weaker response to treatment with imide drugs targeting IKZF1 [229].

Rare variants of the *BCL2* translocation involving the *IGK* [t(2;18)(p11;q21)] or *IGL* [t(18;22)(q21;q11)] loci have been reported in follicular lymphoma (FL) [230,231,232,233,234,235] and chronic lymphocytic leukemia (CLL) [145,236,237]. Cases with these translocations were positive for BCL2 protein expression. Similarly to the variant *MYC* translocations, the breakpoint in *BCL2* was different from that involved in translocations with *IGH*, and was localized at the 5′ end of the *BCL2* gene (Figure 2).

*CCND1* is commonly translocated to *IGH* in mantle cell lymphoma (MCL). Case studies also reported MCL patients with translocations involving *CCND1* or *CCND2* and *IGL* or *IGK* resulting in strong overexpression of cyclin D1 or D2 [238,239,240,241,242,243]. However, in a subset of cyclin D1-negative MCL cases the underlying molecular mechanism of the disease remained unclear. Recently, Martin-Garcia et al. investigated 56 cyclin D1-negative MCL cases using FISH, whole genome/exome sequencing and gene expression arrays. They found *CCND2* or *CCND3* rearrangements in 93% of the cases. Majority (70%) displayed conventional translocations with *IGL* or *IGK*. In a few cases the authors identified cryptic insertions of the *IGK* or *IGL* enhancers close to *CCND2* and *CCND3* genes which led to overexpression of those cyclins. Expression profiles and clinical outcome of cyclin D1^−^ and cyclin D1^+^ MCL cases was similar, indicating that the hijacking of *IGK/IGL* enhancers by *CCND2* and *CCND3* may be a molecular event involved in MCL pathogenesis [244].

Other, less frequent translocations found in B-cell lymphomas involved *IGK/IGL* and *BCL3, BCL6, BCL10* or *REL* or other regions with yet undefined partner genes [138,245,246,247,248,249,250] (Table 3).

### 3.3. Role of IGK and IGL Enhancers in Regulating Oncogene Expression and Malignant Development

Increased expression of respective oncogenes in cell lines and patient samples bearing *IGK* or *IGL* translocations only indirectly indicates the role of immunoglobulin light chain enhancers in driving the expression of translocated genes. Overexpression of constructs mimicking the t(2;8) translocation identified the intronic and 3′ kappa enhancers together with the matrix attachment region (MAR) as the elements necessary and sufficient for high *MYC* transcription and change in *MYC* promoter usage from P2 (predominant in normal cells) to P1 (predominant for the translocated *MYC* allele) [251]. Since activity of iEκ critically depends on binding of NF-κB, and 3′Eκ—on SP1—their role in MYC activation was examined. Joint mutations of the respective binding sites completely abolished transcription from the P1 promoter. Similar effect was observed upon NF-κB depletion, while overexpression of both NF-κB subunit REL65 and SP1 synergistically promoted activity of P1 [252].

Further evidence for the role of *IGK/IGL* enhancers in tumorigenesis comes from mouse models. In parallel with the Eµ-Myc model where *Myc* is coupled with the Eµ *IGH* enhancer, mice mimicking the *IGK-MYC* translocation were generated. The Eκ-SV-Myc mice developed lymphomas, which confirms the role of the iEκ enhancer in lymphomagenesis. However, penetrance was lower and latency was higher compared to the Eµ-Myc mice [199]. Mice carrying the λ-Myc transgene under control of the *IGL* enhancer developed high penetrance lymphomas originating from lymph nodes; they presented the ‘starry sky’ appearance characteristic of BL [253]. This confirms the oncogenic potential of the translocated *IGL* enhancer. Compared to the Eµ-Myc model, λ-Myc mice developed lymphomas with more mature phenotype, closer reminiscent of the human BL. Another model of an *IGK/IGL*-driven malignancy is the mouse plasmacytoma (MPC). The disease is induced by pristine oil, alone or combined with Abelson virus, and is characterized by translocations of *Myc* with immunoglobulin loci. In majority of cases *IGH* is involved but translocations with *IGK* or *IGL* have also been reported [254,255,256]. The MPC model demonstrates that *IGK* and *IGL* are able to drive Myc expression which initiates the disease, although additional genetic lesions may be required for the full onset disease [257].

Altogether, this highlights the importance of immunoglobulin light chain enhancers as alternative drivers of B-cell malignancies, as well as the diagnostic and prognostic potential of detecting *IGK/L* translocations. However, more precise dissection of underlying mechanisms is still pending.

## 4. Enhancer Variants and Mutations in B-Cell Malignancies

Cancers are driven by accumulation of mutations. Moreover, inherited sequence variants can also influence susceptibility to malignant transformation. Whole genome sequencing (WGS) revealed a broad spectrum of recurrent, cancer-specific somatic mutations, while genome-wide association studies (GWAS) identified germline sequence variants associated with cancer risk. Recently, mutations and variants in the non-coding parts of the genome have attracted attention. Several risk loci and driver mutations in non-coding regions have been identified and shown to affect gene expression regulatory networks by e.g., interfering with transcription factor binding, shaping chromatin architecture or affecting miRNA binding to target genes [258,259]. Among them, variation in enhancers has been observed in B-cell malignancies and their functional consequences have been highlighted.

### 4.1. Somatic Mutations

A number of enhancers have emerged so far as mutational hot-spots in several B-cell malignancies (Table 4). WGS analysis of matched tumor-normal tissues from CLL patients revealed, in addition to mutations in protein-coding genes, several somatic mutations in non-coding regions. Among them, an intergenic region at chromosome 9p13 was densely mutated in 11% of cases. This region was enriched in transcription factor binding sites and chromatin marks for active enhancer specifically in B cells. 4C-seq revealed interaction with the *PAX5* locus. CRISPR-introduced specific point mutations in the enhancer or its deletion downregulated PAX5 expression by 40%, confirming the functional significance of mutations. However, the effect of mutations on chromatin architecture or TF binding was not investigated. Somatic mutations in the *PAX5* enhancer were also found by the authors in other types of B-cell lymphoma: DLBCL (29%), FL (23%), MCL (5%) [260]. An independent study focusing on somatic regulatory variants in DLBCL confirmed preferential mutation of the *PAX5* enhancer in 23% of the germinal center B-cell subtype of DLBCL [261]. The *PAX5* enhancer was also mutated in BL, especially in EBV-positive cases [262]. PAX5 is a transcription factor with an important role in B-cell commitment and development. Tight regulation of PAX5 levels is critical for normal B-cell lymphopoiesis but also to prevent tumor development. On one hand, *PAX5* is involved in translocations with *IGH*, which lead to PAX5 upregulation in aggressive B-cell lymphomas. On the other hand, PAX5 was shown to act as a haploinsufficient tumor suppressor in B-ALL [263,264]. So far, the effect of mutations in *PAX5* enhancer was studied only in CLL where the associated decrease in PAX5 expression suggests a tumor suppressor role of PAX5.

Other mutation hot-spots in B-cell lymphoma were the enhancers of *BCL6*, *BCL2* and *ST6GAL1* [261,262,265]. A study focusing on mutations in transcription factor binding sites (TFBS), including the above-mentioned enhancers, in combination with RNA-seq data showed that in general mutations in TFBS are associated with altered gene expression. However, the direct effect of mutations in enhancers on their activity and expression of respective genes remains to be investigated. BCL6 and BCL2 are oncogenes with anti-apoptotic role, often mutated in B-cell malignancies and involved in translocations with immunoglobulin genes [267,268]. ST6GAL1 is involved in protein and lipid glycosylation, its upregulation and oncogenic function was reported in several cancers [269]. Thus, mutations in *BCL2*, *BCL6* and *ST6GAL1* enhancers would be expected to augment their activity.

An alternative approach used data from Hi-Ci in naïve B cells to determine regions interacting with promoters as *cis*-regulatory elements (CREs), which were further sequenced in search for somatic mutations. This revealed 78 recurrently mutated CREs interacting with promoters of 72 genes in DLBCL, and 42 recurrently mutated CREs interacting with promoters of 37 genes in FL. As an example, a mutated CRE enriched in enhancer marks and interacting with the *TPRG1* promoter was further characterized. A mutation in the *TPRG1* enhancer was associated with higher TPRG1 expression in DLBCL. In addition, amplification of *TPRG1* gene was observed as an alternative mechanism of TPRG1 upregulation in DLBCL, implicating its significance in lymphoma. The function of TPRG1 is poorly characterized and requires further investigation [266].

Notably, several of those studies observed that enhancers were enriched in mutations affecting the C in the WRCY motif, which is a signature of AID-induced mutations [259,260,261,262,266]. This is in line with a previous report that AID off-targets at non-immunoglobulin loci are predominantly clustered in super-enhancer regions [115]. Characteristic features of enhancers targeted by AID mutations were active transcription of enhancer RNAs and engagement in long-range chromatin interactions. Analysis of BL and DLBCL tumors revealed that apart from the IG genes, main loci of AID mutations were active enhancers of genes with a known role in lymphoma: *BCL6*, *PAX5*, *ETS1*, *CIITA*, *CXCR4* [115]. This highlights AID as an important, and perhaps major, cause of somatic mutations in enhancers in B cells. A systematic analysis of enhancer mutations in B-cell malignancies could reveal other potential underlying mechanisms.

Although several mutations in enhancers were shown to affect expression of genes relevant for B-cell malignancies, significance of the mutations in tumorigenesis remains to be established. Targeted sequencing of 12 super-enhancers in B cells isolated from healthy individuals revealed ~9000 low frequency mutations in all samples. ~8000 of those were localized in the *BCL6* enhancer with a mutation frequency of 2.2 × 10^−4^; other clusters mapped to the *PAX5* and *CD83* enhancers with a lower frequency (6.9–9.7 × 10^−6^). These mutations were specific for the memory B cells. Again, mutation pattern highlighted the role of AID [270]. A larger-scale study and follow-up of the individuals presenting mutations in enhancers would give insights into their prevalence and penetrance, but it is unlikely that they could lead to malignancy without additional genetic lesions. Similarly, oncogenic *IGH* translocations were observed in blood of up to 25% of healthy donors [90,91,92,93]. They persisted in the B-lymphocyte pool for years without any symptoms of B-cell malignancy, which indicates that additional events are required for lymphomagenesis.

### 4.2. Germline Sequence Variants

GWAS studies identified several risk loci for B-cell malignancies and some follow-up studies revealed that several of them harbor single nucleotide variants (SNVs) within enhancers and super-enhancers (Table 5). Two studies focused on enhancer variants within previously identified risk loci in CLL and identified several features indicating their functional importance [271,272]. Firstly, several enhancer SNPs were located in binding motifs for TFs such as SPI1, NFKB, PAX5, MEF2A, FOXI1, NFATC1 and TCF3, with a potential to disrupt or enhance their binding. Indeed, allelic imbalance was observed in ChIP experiments for several SNPs and TFs. Secondly, altered chromatin accessibility and levels of histone marks such as H3K27ac, H3K4me1 and H3K4me3 were observed for alternative alleles in those SNPs, and for some variant loci H3K27ac signals were significantly higher in CLL than in normal B cells. Thirdly, analysis of chromatin architecture revealed that the enhancers harboring risk SNPs interacted with several genes with established roles in B-cell development and malignancy, e.g., *MYC*, *BCL2*, *BCL6*, *IRF4*, *IRF8*, *BCL2L11*, *CDKNA*, *CDKNB*. Moreover, gene expression QTL analysis revealed risk loci with an effect on gene expression. These studies highlighted the potential role of enhancer variants in B-cell malignancies. It remains to be further investigated to what extent such SNPs can affect chromatin interactions, TF binding and gene expression, and whether there is a direct link with development of B-cell malignancies.

Another study in CLL provided functional insights into a super-enhancer polymorphism at 15q15.1 risk locus. SNP rs539846 C > A is localized in a SE in the intron 3 of *BMF* gene, which encodes a pro-apoptotic member of the BCL2 family. The SNP alters a conserved RELA binding motif and was shown to disrupt RELA binding, reduce enhancer activity, and was associated with decreased BMF expression in primary CLL cases. BMF is a BCL2 antagonist, thus reduced BMF levels together with increased BCL2 expression observed in CLL may cooperate to attenuate apoptosis. Although no associations were found between the rs539846 genotype and prognosis or survival, this study revealed a mechanism underlying the 15q15.1 risk locus in CLL [273].

A follow up of two risk loci for childhood ALL identified previously in a GWAS revealed two SNPs located in enhancers of *BMI1* and *PIP4K2A*. rs11591377 lies in a region showing strong enhancer marks in hematopoietic cells and containing binding sites for multiple transcription factors. This enhancer interacted with the *BMI1* promoter in myeloid and B-cells but not T-cells. The risk G allele was predicted to enhance binding of MYBL2 and p300 transcription factors, which was demonstrated in K562 cells heterozygous for this SNP. Another SNP, rs4748812, was located in an enhancer region interacting with the *PIP4K2A* promoter in B cells. The rs4748812 risk allele T was predicted to create a RUNX1 binding site, but this was not proven experimentally [274].

A thorough functional investigation of a *GATA3* enhancer variant provided insights into B-ALL pathogenesis. rs3824662 located in a region with enhancer features in hematopoietic cells was associated with susceptibility to Ph-like ALL. The risk variant A allele increased activity of the enhancer in a reporter assay and was also associated with higher H3K4me1 mark and open chromatin in B cells. The enhancer formed a chromatin loop with the *GATA3* promoter. Accordingly, GATA3 expression was increased in primary leukemia samples with the risk allele and in a CRISPR-engineered LCL cell line with the A/A genotype, but no effect was observed on expression of other genes in the topologically associated domain. A binding site for the transcription factor NFIC was identified in the vicinity of the variant and ChIP confirmed stronger binding of NFIC to the A allele. Globally, the A allele induced binding of GATA3 to novel sites genome-wide and changes in the 3D genome organization and gene expression profile. An interesting observation was made that GATA3 binding motif was enriched near breakpoint regions in Ph-like ALL, which suggests that GATA3 may be involved in this translocation [275]. It would be interesting to investigate whether noncoding transcription at these loci may contribute to the rearrangements, as is the case for *IGH* translocations.

An integrative analysis of FAIRE-seq and histone marks ChIP-seq revealed distal regulatory elements (DREs) which differed in activity between follicular lymphoma samples and normal centrocytes. The variable DREs were enriched for SNPs and SNVs predicted to disrupt TF binding motifs. Three sequence variants, in BS for IKZF1, SP1 and TCF3, were further investigated. All three variants reduced binding of respective TFs and decreased enhancer activity. Analysis of gene expression in FL samples revealed that predicted target genes of these TFs were downregulated in FL samples with the sequence variants. These included several genes which have been associated previously with B-cell malignancies (*HLA-DQA1*, *DUSP6*, *IRF8*) [276].

In summary, available data highlight the significance of somatic mutations and germline variants in enhancers as another mechanism of enhancer repurposing in B-cell malignancies. Functional studies revealed a profound impact of enhancer mutations and SNPs on chromatin architecture, TF binding and expression of genes involved in normal and pathological processes in B cells. Given the large number of non-coding mutations and variants observed in tumors and GWAS studies, more insights into the role of enhancer variants in B-cell malignancies are expected.

## 5. Exploiting Enhancers by Deregulated Transcription Factors

Enhancers are packed with transcription factors (TF) motif sequences. TF binding indicates active enhancer regions and is necessary for target genes activation [277]. In cancer cells, TF expression is often altered, which in consequence leads to aberrant binding at enhancers and ultimately changes expression of the controlled genes [278,279]. Here we describe a few examples of how deregulated TFs rewire enhancers’ activity in B-cell neoplasms.

Sequential activation of the PAX5 transcription factor determines the B-cell commitment in early stages of lymphopoiesis. B-cell specific expression of PAX5 is controlled by several TFs (PU.1, IRF4, IRF8 and NF-κB) binding to an enhancer in intron 5 of *PAX5* [280]. Thus, deregulation of those TFs, which occurs in B-cell malignancies, affects expression of PAX5. Furthermore, PAX5 itself regulates expression of several target genes in B cells by rapidly recruiting chromatin modifying proteins to their promoters and enhancers. Presence of PAX5 on chromatin correlated with increased active chromatin marks in PAX5-induced genes, whereas an inverse pattern of histone modifications was observed in PAX5-repressed genes [281]. As demonstrated later, another B-cell specific transcription factor, EBF1, is required for the interaction of PAX5 with the MLL H3K4 methyltransferase complex and subsequent epigenetic modifications [282]. EBF1 and PAX5 have opposing roles in normal and malignant B cells with regards to the regulation of the *MYC* oncogene. Both EBF1 and PAX5 are bound to *MYC* enhancers in mouse pro-B cells as well as pro-B ALL NALM6 cells. While EBF1 promoted MYC expression, PAX5 negatively regulated MYC levels in normal B-cell progenitors [283]. Although it is not clear how this regulation looks in malignant cells, another report suggested that EBF1 and PAX5 prevent malignant transformation by limiting MYC levels [284].

Another transcription factor with a crucial role in hematopoiesis is RUNX1. Mutations and translocations involving *RUNX1* are frequent in hematologic malignancies [285]. In human pre-B leukemia cells RUNX1 together with FUBP1 bound to an intronic enhancer in the oncogene *c-KIT*. Overexpression of RUNX1 and FUBP1 upregulated c-KIT levels and enhanced cell proliferation, as well as decreased cell sensitivity to the c-KIT inhibitor and therapeutic drug imatinib mesylate [286]. RUNX1 also interacts with CBFA2T3 which enhances its transcriptional activity. They act in a self-activation loop, as RUNX1 binds its own promoter and the *CBFA2T3* enhancer located 2 kb upstream of the *CBFA2T3* promoter [287]. Since RUNX1 and CBFA2T3 are upregulated in ETV6-RUNX1 B cell precursor ALL (BCP-ALL) [288], it suggests that RUNX1 and CBFA2T3 may act as a driver loop in BCP-ALL. Indeed, use of a truncated CBFA2T3 protein significantly inhibited RUNX1 activity and reduced BCP-ALL cell proliferation [287].

The chimeric transcription factor TCF3-HLF, resulting from the t(17;19)(q22;p13) translocation, is associated with poor survival and resistance to therapy in B-ALL [289]. ChIP-Seq in leukemia cells revealed prevalent binding of TCF3-HLF to active enhancers, especially super-enhancers. Among them was a distal *MYC* SE possessing a HLF binding motif. CRISPR-mediated disruption of the HLF motif disturbed interactions between the SE and the *MYC* promoter, reduced MYC expression and decreased viability of HAL-01 cells. The activating effect of TCF3-HLF on enhancers was mediated by the recruitment of the p300 acetyltransferase and was thus vulnerable to an inhibitor of p300, A-485 [290].

MEF2B is a transcription factor often mutated in DLBCL and FL, which leads to its increased activity and upregulation of one of its target genes, *BCL6* [291]. ChIP-Seq revealed enrichment of MEF2B and the p300 acetyltransferase at *BCL6* super-enhancer. It was demonstrated that MEF2B directly activates BCL6 expression by increasing histone acetylation at its enhancer [292]. Similarly, activation of BCL2 is observed in MLL-rearranged leukemia patients [293]. The MLL-AF4 fusion protein resulting from the t(4;11)(q21;q23) translocation was shown to bind to the *BCL2* enhancer, consisting of two H3K27Ac clusters at the 3′ end of the gene. The authors demonstrated that MLL-AF4 regulates BCL2 expression by controlling H3K27Ac levels at its enhancer [294].

Global H3K27ac HiChIP analysis identified multiple interactions between enhancers and promoters in several primary effusion lymphoma (PEL) cell lines. In particular, super-enhancers of *MYC* and *IRF4* were critical for PEL cell growth. Transcription factors MEF2C and IRF4 bound to these SE and controlled expression of MYC and IRF4 by promoting H3K27ac. In addition, a global reduction in H3K27ac signals was observed upon CRISPR inactivation of the *IRF4* SE, which suggests that *IRF4* SE and IRF4 are master regulators of the enhancer landscape in PEL cells [295].

These studies demonstrate that physiological interactions between TFs and enhancers, essential for proper B-cell development and function, may become pathogenic upon dysregulation of TF levels.

## 6. Enhancer Hijacking by Lymphoma-Associated Viruses

Certain viruses have been implicated in B-cell malignancies, e.g., Epstein–Barr virus (EBV), Kaposi’s sarcoma-associated herpesvirus (KSHV), human immunodeficiency virus (HIV), hepatitis C virus (HCV). Viruses rely on the host factors for their own replication and have mastered the ability to reprogram the host cell transcription and translation machinery as well as metabolism for their own purpose. One of the mechanisms exploited by viruses is hijacking host cell enhancers to change the epigenetic landscape and to promote a gene expression profile that creates a favorable environment for virus replication.

### 6.1. Epstein–Barr Virus

The best studied virus associated with B-cell lymphomas is Epstein–Barr virus (EBV). EBV is a human gamma-1 herpesvirus that shows tropism for B cells and is commonly present in the latent form in >90% of worldwide population. While majority of carriers are asymptomatic, in some cases infectious mononucleosis can develop. EBV has been also associated with B-cell malignancies: eBL, cHL and DLBCL. Endemic Burkitt lymphoma is a canonical example of EBV-linked malignancy. Virtually all cases of eBL are positive for EBV infection. Given the widespread persistence of EBV in the population, clearly EBV infection alone is not sufficient for lymphomagenesis. Compromised immune response, e.g., in case of malaria, AIDS or in post-transplantation patients releases EBV-infected cells from immune surveillance by T cells and increases risk of malignant transformation [296]. In vitro infection of B lymphocytes with EBV causes their immortalization and establishment of continuously proliferating lymphoblastoid cell lines (LCLs). A wide set of viral proteins is involved in B-cell immortalization but only a few are expressed later in the latent state, depending on the latency type (e.g., EBNA2, EBNA3 and EBNALP proteins). While the association of EBV with certain types of B-cell lymphomas is undisputable, still its precise role and mechanisms behind EBV-linked lymphomagenesis are not fully understood [297]. Recently, enhancer hijacking by EBV resulting in subsequent chromatin reorganization and transcriptional reprogramming has been highlighted in several studies.

Zhou et al. provided a global overview of EBV-controlled enhancers in a lymphoblastoid cell line GM12878. EBNA2-ChIP-seq identified 888 sites with very strong EBNA2 binding and high and broad H3K27ac signals, characteristic of super-enhancers (SEs). EBNA2 SEs were often localized near genes encoding essential B-cell TFs (e.g., MYC, MAX, RUNX3), and were often co-occupied by other B-cell TFs (e.g., ETS1, IRF4, SPI1, STAT5, PAX5). RBPJ, a TF which often mediates binding of EBNA proteins to DNA, was also found in many of those sites. Apart from EBNA2, viral oncoproteins EBNA3A, EBNA3C and ENBALP are also involved in regulating gene expression in EBV-infected cells. Moreover, NF-κB is essential for LCLs survival. Thus, the authors searched for SEs with co-occupancy of all four oncogenic EBNAs and five NF-kB subunits. 187 such sites were identified and designated as EBV SEs. Genes associated with EBV SEs included *MYC*, *BCL2*, *RUNX3*, *IKZF3*, oncomiRs miR-155, miR-21 and let-71, and were involved in apoptosis, DNA damage repair and MAPK signaling. IGL enhancer was also occupied by EBNA [298].

Hijacking the *MYC* enhancer by EBV has been extensively studied. A region spanning 428-556 kb 5′ of *MYC* was strongly bound by EBNA2 an RBPJ and possessed features characteristic of active enhancers: high H3K4me1, H3K9ac, RNAPII and p300 signals. FISH assay with probes for the *MYC* promoter and distal enhancer confirmed their interaction. EBNA2 inactivation significantly diminished colocalized signals, indicating that the association of *MYC* enhancer and promoter depends on EBNA2 [299]. EBNA2-dependent loop formation between the *MYC* SE and promoter was confirmed later by chromosome conformation capture [300,301] and RNAPII ChIA-PET [302]. Importance of the *MYC* SE for EBV-infected cells was proved by reduced MYC expression and cell proliferation upon CRISPR/Cas9-mediated deletion of the SE [302]. Moreover, eRNAs transcribed from EBV SEs, including the *MYC* SE, were identified. Expression of *MYC* SE eRNAs was dependent on EBNA2, and their knockdown inhibited proliferation of LCLs, decreased MYC expression, and reduced H3K27ac signal and looping of *MYC* SE to promoter [300]. Altogether, EBV rearranges chromatin architecture in the *MYC* locus to promote its expression and proliferation of EBV-infected cells.

EBNA2 and EBNA3 proteins (3A and 3C) target common sites and genes. Majority of sites bound by EBNA2 and 3 carried histone marks characteristic for active enhancers: high H3K27ac and H3K4me1, while some were poised enhancers (H3K27ac−, H3K4me1+). However, Re-ChIP analysis revealed that EBNA2 and 3 do not bind simultaneously to the same sites, they are exclusive [303]. While EBNA2 is an activator of transcription, EBNA3 can act as both an activator and a repressor. Binding of EBNA2 and 3 to several enhancers was shown to affect genes crucial for B-cell survival, and in some instances the two EBNA proteins counteracted each other. Distant enhancers upstream and downstream of *BCL2L11* gene form loops with the *BCL2L11* promoter in EBV-negative cells, and these interactions are lost upon EBV infection. It has been shown that EBNA3A and 3C bind to those enhancers and disrupt looping with promoter by recruiting the PRC complex which deposits the silencing mark H3K27me3 across the *BCL2L11* promoter [301]. As a result, the pro-apoptotic BIM protein encoded by *BCL2L11* is repressed, which counteracts the MYC-induced apoptosis. Similar mechanism of EBNA3 and PRC-mediated disruption of chromatin interactions and repression of transcription was observed for the *CDKN2A/B* loci encoding the tumor suppressors p16INK4a, p15INK4b and p14 ARF [302].

Interplay between EBNA2 and 3 proteins affecting B-cell growth was revealed for RUNX transcription factors [304]. SE of *RUNX3* is bound by EBNA2, EBNA3A and EBNA3C which cooperatively promote RUNX3 expression in an RBPJ-dependent way. RUNX3 is required for proliferation of LCLs and was previously shown to negatively regulate expression of RUNX1 [305]. In EBV-positive BL cells, but not LCLs, *RUNX1* enhancer was also bound by EBNA2, which resulted in activation of RUNX1 expression. However, this effect was attenuated by EBNA3B and C which also bound *RUNX1* SE and repressed its expression [304]. Why EBNA2 activates RUNX1 in some EBV-positive cells and not in others requires further investigation. Possible role of MYC has been suggested as well.

An interesting link between EBV and somatic hypermutation in the immunoglobulins has been discovered by Kalchschmidt et al. They observed increased levels of AID mRNA and protein driven by EBNA3C. Furthermore, ChIP revealed EBNA3C occupancy at the SE of *AICDA* gene encoding AID. Again, binding of EBNA3C depended on the interaction with RBPJ. Increased levels of histone marks characteristic for enhancers, H3K4me3, H3K9ac, and H3K27ac, as well as recruitment of p300 to the *AICDA* SE was observed only in the presence of functional EBNA3C. Importantly, EBNA3C-induced AID was functional and caused SHM in the V(D)J region of IGH [306]. In the light of the well-documented off-target AID activity in non-Ig genes which promotes translocations between Ig loci and oncogenes, this study provides a possible link between EBV and lymphomagenesis.

EBNA2 and 3 proteins have been also implicated in regulation of some oncogenic miRNAs. miR-221 and miR-222 are expressed from one pri-miR and they are often upregulated in several cancers, including DLBCL. In EBV-positive cells expression of mature and pri-miR-221/222 was regulated by EBNA3A and 3C. ChIP and chromosome conformation capture analyses revealed that this activation is mediated by EBNA3A and 3C binding to an enhancer upstream of miR-221/222 cluster, which leads to increased levels of active chromatin marks and looping between the enhancer and promoter. P57^KIP2^, a negative regulator of cell proliferation, was validated as a target of miR-221/222. However, inhibition of miR-221/222 and subsequent upregulation of P57^KIP2^ had only a mild effect on LCL cells proliferation, indicating that other targets of miR-221/222 may be relevant [307]. miR-155 is involved in normal hematopoiesis and overexpressed in B-cell lymphoma (HL, DLBCL). miR-155 was also upregulated in B cells upon EBV infection. EBNA2 was shown to promote expression of miR-155 two-way. First, directly by RBPJ-mediated binding to an enhancer upstream of the miR-155 host gene. Second, indirectly by RBPJ-mediated binding to an *IRF4* enhancer. IRF4 binds to the same miR-155 enhancer, thus additionally boosting miR-155 expression [308].

**Figure 4:**
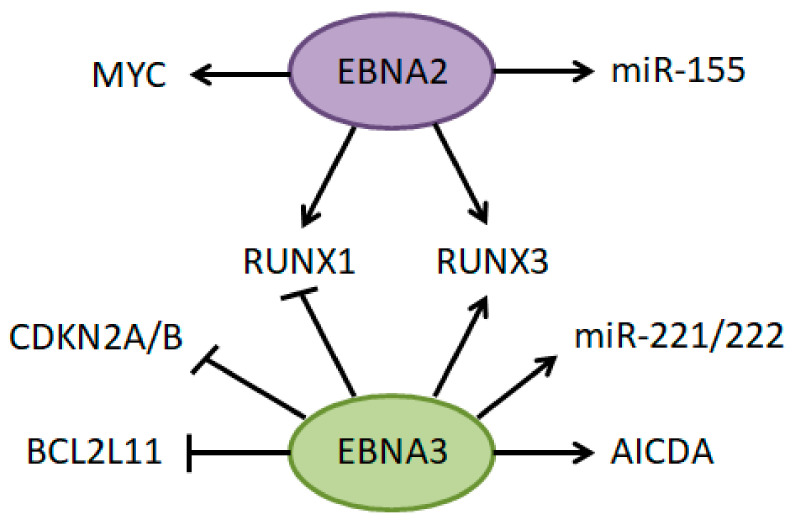
Enhancer hijacking by Epstein–Barr virus. Presented are interactions of Epstein–Barr Virus Nuclear Antigen 2 and 3 (EBNA2 and EBNA3) proteins with host gene enhancers. Arrows indicate activation of gene expression; bars represent inhibition.

Taken together, these data indicate how hijacking cellular enhancers by EBV promotes B-cell proliferation and can contribute to lymphomagenesis (Figure 4). EBV upregulates MYC which boosts cell proliferation. At the same time, expression of the pro-apoptotic protein BIM is downregulated, counteracting the MYC-induced apoptosis. Increased activity of *MYC* enhancers can also promote translocations as it has been demonstrated that sites of active non-coding transcription are hotspots for AID-induced breakpoints [115,116]. In line with this, breakpoints in eBL are located in the 5′ distal region of *MYC*, in contrast to sporadic BL where they are mostly located within the *MYC* gene body. In addition, EBV also induces expression of AID, further promoting translocations. Since EBV-positive lymphomas do not express EBNA2 and 3 proteins, events described above are likely to contribute to development of lymphomas rather than maintaining established tumors.

### 6.2. Kaposi’s Sarcoma-Associated Herpesvirus

Another virus involved in pathogenesis of B-cell malignancies is Kaposi’s sarcoma-associated herpesvirus (KSHV) which causes primary effusion lymphoma (PEL). PEL is a rare, aggressive disease occurring in immunocompromised patients. 60–90% of PEL cases are also positive for EBV [309]. In KSHV-infected cells the virus is maintained in a latent state with only a few viral genes expressed that sustain cell proliferation. Lytic state is activated in a subset of cells to allow virus replication. A master host transcription factor essential for PEL cells is IRF4 which binds to enhancers and drives expression of e.g., MYC and BATF3 [310]. Viral interferon regulatory factor 3 (vIRF3) was shown to associate with IRF4 and BATF at active enhancers to promote expression of several genes essential for PEL cells. Lack of either IRF4 or vIRF3 resulted in decreased enhancer activity. Over 60% of PEL essential genes were downregulated upon knockout of IRF4, BATF or vIRF3. Gene set enrichment analysis indicated MYC targets and cell cycle genes among genes regulated by IRF4 and vIRF3, which implies important function of KSHV in proliferation of PEL cells [311]. However, it is unclear how IRF4 and vIRF3 get hold of enhancers in PEL cells, e.g., whether vIRF3 and IRF4 shape chromatin architecture themselves or is their binding to enhancers facilitated by chromatin opening by other factors.

Another study performed a global analysis of epigenetic marks and nascent transcription in KSHV-positive PEL cells during virus latency and upon lytic reactivation. This revealed that during latency, super-enhancers for several oncogenes, including *MYC*, are activated by KSHV and repressed upon transition to the lytic state. GRO-seq confirmed that lytic reactivation resulted in a widespread shutdown of host gene transcription, including eRNAs. Further insights were gained into the regulation of MYC, which was previously shown to maintain KSHV latency and proliferation of PEL cells [312]. Strikingly, in PEL cells active enhancer marks and eRNA transcription were observed ~500 kb downstream of *MYC*, in contrast to EBV-infected cells where the active enhancer was located upstream of *MYC*. 4C experiments confirmed interaction of the downstream enhancer with *MYC* promoter in PEL cells, and CRISPRi targeting of the enhancer or eRNA inhibition reduced MYC expression and activated the lytic state. However, the role of viral proteins in the enhancer activation in latent state was not studied. Instead, it was shown that the host IRF4 activates the *MYC* enhancer during KSHV latency and upon viral reactivation the viral vIRF4 represses the cellular IRF4 leading to MYC repression [313].

Altogether, the data so far clearly highlight the hijacking of cellular enhancers by viruses as an important mechanism in B-cell lymphomagenesis. Given the limited repertoire of viral proteins, this is an efficient way to ensure proliferation of the host cells together with the virus and lytic reactivation to produce viral progeny. Genes controlled by the viruses for the sake of increased proliferation have often oncogenic properties and thus enhancer hijacking explains some aspects of the role of viruses in B-cell lymphomas.

## 7. Conclusions and Future Perspective

Cancer can be viewed as a disease of the genome caused by accumulation of acquired and hereditary alterations in the DNA. Recent advances clearly indicate that the non-coding, regulatory parts of the genome are critically involved in cancer pathogenesis. He we presented an overview of the role of enhancers in B-cell malignancies. Studies have demonstrated a variety of mechanisms through which enhancers controlling gene expression for proper B-cell development can be repurposed to direct the cell on a path toward malignant transformation. The emerging role of enhancers in the pathogenesis of B-cell malignancies marks a shift in cancer research: instead of paying attention to the ingredients that make up a malignant cell, focusing on the cook who determines their proportions.

Apart from broadening our understanding of B-cell malignancies and highlighting the role of non-coding sequences, this knowledge can also provide novel directions for therapeutic options. General enhancer inhibitors like BET-bromodomain protein inhibitor JQ1 or HDAC inhibitors have been investigated in different tumors [314,315]. Given the fundamental role of IGH enhancers in lymphomagenesis, they appear as attractive targets for therapeutic approaches [190,316]. Although disruption of *IGH* regulatory elements will likely affect normal B cells, transient impairment of humoral immune response is well-tolerated in humans as has been shown using the B-cell eradicating anti-CD20 antibody Rituximab that is commonly used for the treatment of B-cell lymphoma. So far, a limited number of compounds inhibiting the activity of *IGH* enhancers have been reported [317,318,319]. Further investigation of specific enhancers and mechanisms through which they are exploited by cancer cells can aid development of novel therapies. Cell-type specific activity of enhancers holds a promise for more precise targeting opportunities.

## Figures and Tables

**Table 1:** Translocations involving immunoglobulin heavy chain locus in B-cell malignancies.

Genes Involved	Translocation	Disease	Consequences	References
*BCL2*	t(14;18)(q32;q2)	90% FL	Delayed apoptosis and accumulation of aberrant cells	[132,133,134,135]
		15–30% DLBCL		
*BCL3*	t(14;19)(q32;q13)	CLL, NHL	Modulation of the NF-kB pathway	[136,137,138,139]
*BCL6*	t(3;14)(q27;q32)	30% DLBCL	Increased cell proliferation, block of terminal differentiation	[140,141,142,143,144,145,146,147]
		4-14% FL		
*BCL10*	t(1;14)(p21;q32)	5% MALT	Activation of the NF-kB pathway (translocation involves a mutant BCL10 which lost pro-apoptotic functions)	[148,149,150]
*CCND1* *(BCL1)*	t(11;14)(q13;q32)	95% MCL	Accelerated passage through the G1 phase	[151,152,153,154,155,156]
		15–20% MM		
		B-PLL, PCL, SLVL		
*CEBPA*	t(14;19)(q32;q13)	ALL	Deregulated cellular proliferation and differentiation	[157,158,159,160]
*CEBPB*	t(14;20)(q32;q13)			
*CEBPD*	t(8;14)(q11;q32)			
*CEBPE*	t(14;14)(q11;q32)			
*CEBPG*	t(14;19)(q32;q13)			
*FGFR3/MMSET*	t(4;14)(p16;q32)	10% MM	Increased cell proliferation and survival	[161,162,163,164,165,166]
*FOXP1*	t(3;14)(p14;q32)	10% MALT	Enhanced tumor cell survival	[167,168,169]
		DLBCL		
*IL3*	t(5;14)(q31;q32)	ALL	Increased cell proliferation and survival	[170,171]
*MAF*	t(14;16)(q32;q23)	MM	Increased cell proliferation	[172,173,174,175]
*MALT1*	t(14;18)(q32;q21)	15–20% MALT	Activation of the NF-kB pathway	[167,176,177]
*MYC*	t(8;14)(q24;q32)	70% BL	Increased cell proliferation	[135,178,179,180,181,182,183]
		ALL		
		DLBCL		
*NFKB2*	t(10;14)(q24;q32)	SMZL	Constitutional activation of the non-canonical NF-kB pathway	[184,185,186]
		MM, CLL		
*PAX5*	t(9;14)(p13;q32)	50% LPL	Dysregulation of PAX5 target genes	[187,188]

ALL, acute lymphoblastic leukemia; BL, Burkitt lymphoma; B-PLL, B-prolymphocytic leukemia; CLL, chronic lymphocytic leukemia; DLBCL, diffuse large B-cell lymphoma; FL, follicular lymphoma; LPL, lymphoplasmacytic lymphoma; MALT, mucosa-associated lymphoid tissue; MCL, mantle cell lymphoma; MM, multiple myeloma; NHL, non-Hodgkin lymphoma; PCL, plasma cell leukemia; SLVL, splenic lymphoma with villous lymphocytes; SMZL, splenic marginal zone B-cell lymphoma.

**Table 2:** Mouse models—*IGH*.

Gene	Translocation	Enhacer Involved	Model Name	Disease	References
*BCL2*	t(14;18)(q32;q21)	3′RR	Igh-3′E-bcl2	FL	[194]
*BCL6*	t(3;14)(q27;q32)	Eμ	Eμ-tTA-BCL6	DLBCL, TL	[195]
*BCL10*	t(1;14)(p22;q32)	Eμ	Eμ-BCL10	MZL	[96]
*CCND1*	t(11;14)(q13;q32)	Eμ	Eμ-CCND1	no *	[196,197]
		3′RR	CCND1-3′RR	no	
*MAF*	t(14;16)(q32;q23)	Eμ	Eμ-c-MAF	MM	[198]
*MYC*	t(8;14)(q24;q32)	Eµ	Eμ-myc iMycEμ	BL	[189,190,191,199,200,201,202,203,204,205]
		Eµ + 3′RR	iMycCα		
		3′RR	IgH-3′E-myc, minimal 3′RR, iMycCµ		

* malignant transformation occurred when crossed with Eµ-myc mice; BL, Burkitt lymphoma; DLBCL, diffuse large B-cell lymphoma; FL, follicular lymphoma; MM, multiple myeloma; MZ, marginal zone lymphoma; TL, T-cell lymphoma.

**Table 3:** Translocations involving immunoglobulin light chain loci in B-cell malignancies.

Gene	IG Light Chain	Translocation	Disease	References
*BCL2*	lambda	t(18;22)(q21;q11)	CLL, FL	[145,230,231,232,233,234,235,236,237]
	kappa	t(2;18)(p11;q21)		
*BCL3*	lambda	t(19;22)(q13;q11)	FL, DLBCL	[138]
	kappa	t(2;19)(p12;q13)	HL, B-cell NHL	
*BCL6*	lambda	t(3;22)(q27;q11)	B-cell NHL	[247,250]
	kappa	t(2;3)(p11;q27)		
*BCL10*	kappa	t(1; 2)(p22; p12)	MALT	[248,249]
*CCND1*	lambda	t(11;22)(q13;q11)	MCL	[238,240,242,243]
	kappa	t(2;11)(p11;q13)		
*CCND2*	lambda	t(12;22)(p13;q11)	MCL	[239,241,244]
	kappa	t(2;12)(p11;p13)		
*CCND3*	lambda	t(6;22)(p21;q11)	MCL	[244]
	kappa	t(2;6)(p11;p21)		
*MYC*	lambda	t(8;22)(q24;q11)	ALL, BL, DLBCL, MM	[223,224,225,226,227,229]
	kappa	t(2;8)(p11;q24)		
*REL*	lambda	t(2;22)(p16;q11)	HL	[245]

ALL, acute lymphoblastic leukemia; BL, Burkitt lymphoma; CLL, chronic lymphocytic leukemia; DLBCL, diffuse large B-cell lymphoma; FL, follicular lymphoma; HL, Hodgkin lymphoma; MALT, mucosa-associated lymphoid tissue; MCL, mantle cell lymphoma; MM, multiple myeloma; NHL, non-Hodgkin lymphoma.

**Table 4:** Somatic mutations in enhancer regions identified in B-cell malignancies.

Gene	Disease	Effect on Gene Expression	Reference
*BCL2*	DLBCL	ND	[261,265]
*BCL6*	BL, DLBCL	ND	[261,262,265]
*PAX5*	BL, CLL, DLBCL, FL, MCL	Decreased	[260,261,262]
*ST6GAL1*	BL	ND	[262,265]
*TPRG1*	DLBCL	Increased	[266]

BL, Burkitt lymphoma; CLL, chronic lymphocytic leukemia; DLBCL, diffuse large B-cell lymphoma; FL, follicular lymphoma; MCL, mantle cell lymphoma.

**Table 5:** Germline variants in enhancer regions associated with B-cell malignancies.

Gene	SNP ID	Disease	Gene Expression	TF Binding	Reference
*BMF*	rs539846	CLL	Decreased	RELA (disrupted)	[273]
*BMI1*	rs11591377	ALL	ND	MYBL2, p300 (enhanced)	[274]
*GATA3*	rs3824662	ALL	Increased	NFIC (enhanced)	[275]
*PIP4K2A*	rs4748812	ALL	ND	RUNX1 (enhanced)	[274]

ALL, acute lymphoblastic leukemia; CLL, chronic lymphocytic leukemia.
